# Novel Reassortant Influenza A(H5N8) Viruses, South Korea, 2014

**DOI:** 10.3201/eid2006.140233

**Published:** 2014-06

**Authors:** Youn-Jeong Lee, Hyun-Mi Kang, Eun-Kyoung Lee, Byung-Min Song, Jipseol Jeong, Yong-Kuk Kwon, Hye-Ryoung Kim, Kyu-Jun Lee, Mi-Seon Hong, Il Jang, Kang-Seuk Choi, Ji-Ye Kim, Hyun-Jeong Lee, Min-Su Kang, Ok-Mi Jeong, Jong-Ho Baek, Yi-Seok Joo, Yong Ho Park, Hee-Soo Lee

**Affiliations:** Animal and Plant Quarantine Agency, Anyang, Gyeonggi, South Korea

**Keywords:** influenza, influenza virus, viruses, highly pathogenic avian influenza virus, HPAI, reassortant, outbreak, H5N8 subtype, South Korea

**To the Editor:** Highly pathogenic avian influenza (HPAI) viruses have caused considerable economic losses to the poultry industry and poses potential threats to animal and human health (www.oie.int/en/ and www.who.int/en/). Since 2003, influenza A(H5N1) viruses with a hemagglutinin (HA) gene derived from A/goose/Guandong/1/96–like viruses have become endemic to 6 countries (Bangladesh, China, Egypt, India, Indonesia, and Vietnam) (*1*) (www.cdc.gov/). Furthermore, HPAI viruses with an H5 subtype continue to undergo substantial evolution because of extensive genetic divergence and reassortment between other subtypes of influenza viruses. Especially in China, novel subtypes of H5 HPAI virus, such as influenza A(H5N2), influenza A(H5N5), and influenza A(H5N8) viruses, were reported during 2009–2011 (*2*,*3*).

On January 16, 2014, clinical signs of HPAI, such as decreased egg production (60%) and slightly increased mortality rates, were detected in ducks on a breeder duck farm near the Donglim Reservoir in Jeonbuk Province, South Korea. On January 17, a farmer (5 km from the Donglim Reservoir) also reported clinical signs of HPAI in breeder ducks. In addition, 100 carcasses of Baikal teals were found in the Donglim Reservoir.

RNAs extracted from organs (liver, pancreas, and trachea) of 3 dead birds (1 breeder duck, 1 broiler duck, and 1 Baikal teal) were positive for H5 subtype virus by reverse transcription PCR (*4*). We isolated viruses from suspected specimens by inoculation into embryonated specific pathogen–free chicken eggs. The H5N8 subtype was identified by using HA and neuraminidase (NA) inhibition assays. 

Three viruses isolated from domestic ducks and wild birds were designated A/breeder duck/Korea/Gochang1/2014 (H5N8) (Gochang1), A/duck/Korea/Buan2/2014 (H5N8) (Buan2), and A/Baikal Teal/Korea/Donglim3/2014 (H5N8) (Donglim3). All 8 RNA genome segments of these viruses were amplified by using segment-specific primers and directly sequenced (*5*). Sequences of the 8 RNA segments of each virus were submitted to GenBank under accession nos. KJ413831–KJ413854.

Gochang1 virus has been shown to be highly pathogenic for chickens (intravenous pathogenicity index 3.0) (*6*). This finding was consistent with analysis of the HA gene, as shown by a series of deduced basic amino acid sequences (Gochang1, LREKRRKR/GLF, Buan2 and Donglim3, LRERRRKR/GLF) at cleavage sites of HA (*6*). This outbreak of influenza A(H5N8) infection in South Korea was reported to the World Organisation for Animal Health (*7*).

Nucleotide identity analysis with BioEdit version 7.2.5 (http://bioedit.software.informer.com/) and ClustalW (www.ebi.ac.kr/Tolls/clustalw2) showed that 3 distinct novel influenza A(H5N8) viruses emerged in South Korea. Gochang1 virus had 87%–97% sequence identities in the 8 genome segments with sequences for Buan2 and Donglim3 viruses, which had high sequence identities (>99.5%) with each other. Conservative amino acid residues within receptor binding pockets of HA (including E190, R220, G225, Q226, and G228; H3 numbering) were present in all 3 viruses, which indicated that these viruses retained affinity for the avian (sialic acid-2,3-NeuAcGal) cell surface (*8*). Although there was an I314V mutation in the NA of the 3 viruses, other mutations that encode oseltamivir and zanamivir resistance were not detected (*9*).

A BLAST (www.ncbi.nlm.nih.gov/genomes/FLU/FLU.html) search and phylogenetic analysis showed that these novel H5N8 subtype viruses likely originated from reassortment between A/duck/Jiangsu/k1203/2010 (H5N8) virus and other subtypes of avian influenza virus, all of which co-circulated in birds in eastern China during 2009–2012 (*10*). A phylogenetic tree of partial HA gene sequences for the 3 virus isolates from South Korea and other H5 subtype viruses (n = 72), showed that Gochang1, Buan2, and Donglime3 belong to the proposed H5 clade 2.3.4.6 (Figure) (*10*).

**Figure:**
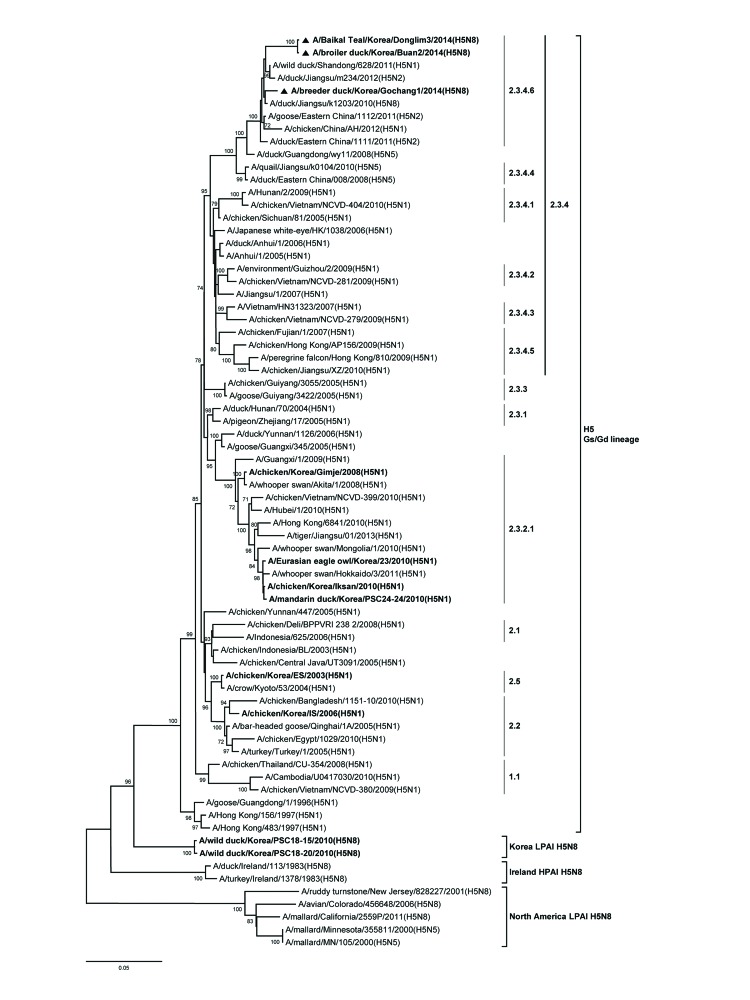
Phylogenetic tree of hemagglutin (HA) genes of influenza A(H5N8) viruses, South Korea, 2014. Triangles indicate viruses characterized in this study. Other viruses detected in South Korea are indicated in boldface. Subtypes are indicated in parentheses. A total of 72 HA gene sequences were ≥1,600 nt. Multiple sequence alignment was performed by using ClustalW (www.ebi.ac.kr/Tolls/clustalw2). The tree was constructed by using the neighbor-joining method with the Kimura 2-parameter model and MEGA version 5.2 (www.megasoftware.net/) with 1,000 bootstrap replicates. H5, hemagglutinin 5; Gs/Gd, Goose/Guangdong; LPAI, low pathogenic avian influenza; HPAI, highly pathogenic avian influenza. Scale bar indicates nucleotide substitutions per site.

The H5 and N8 genes of the 3 viruses had high nucleotide identities with A/duck/Jiangsu/k1203/2010 (H5N8) (JQ97369691–98) (H5: Gochang1, 98.9%, Buan2 and Donglim3, 97.2%; N8: Gochang1, 98.5%, Buan2 and Donglim3, 98.1%). For Gochang1 virus, polymerase basic protein 2 (PB2) and nonstructural (NS) protein had the highest identities with A/environment/Jiangxi/28/2009 (H11N9) (PB2 98.6%, NS 97.7%). The other segments showed high genetic identities with A/duck/Jiangsu/k1203/2010 (H5N8) (>98.7%), which suggested that Gochang1 virus was generated by reassortment in which the PB2 and NS genes of A/duck/Jiangsu/k1203/2010 (H5N8) were replaced by those of influenza A(H11N9) viruses.

For Buan2 and Donglim3 viruses, the PB2, HA, nucleoprotein, and NA genes were highly similar to those of A/duck/Jiangsu/k1203/2010 (H5N8) (>97.2%). However, the PB1, polymerase acidic protein, matrix protein, and NS genes of this virus had the highest genetic identities with A/duck/Eastern China/1111/2011 (H5N2) (>98.2%). Therefore, Buan2 and Donglim3 viruses might be reassortants that contain PB2, HA, nucleoprotein, and NA genes from A/duck/Jiangsu/k1203/2010 (H5N8) and PB1, polymerase acidic protein, NS, and matrix genes from A/duck/Eastern China/1111/2011 (H5N2) co-circulating in the same region of China (*2*,*10*).

We characterized 3 distinct novel reassortant influenza A(H5N8) HPAI viruses during an influenza outbreak in South Korea. Buan2 and Donglim3 viruses showed high nucleotide identities, which suggested that the outbreak viruses in domestic ducks and Baikal teals might have an identical origin. Although research on the epidemiologic features of this outbreak is currently underway, it seems likely that on the basis of reassortant sequence features of the 8 genome segments, the 3 distinct viruses originated in eastern China. These influenza viruses are a potential threat to the poultry population in South Korea, including gallinaceous birds, during movement of domestic ducks through the distribution network of live bird markets.

## References

[1] Alexander DJ, Brown IH. History of highly pathogenic avian influenza. Rev Sci Tech. 2009;28:19–38 . 19618616 10.20506/rst.28.1.1856

[2] Zhao G, Gu X, Lu X, Pan J, Duan Z, Zhao K, Novel reassortant highly pathogenic H5N2 avian influenza viruses in poultry in China. PLoS ONE. 2012;7:e46183. 10.1371/journal.pone.0046183 23049973 PMC3458027

[3] Zhao K, Gu M, Zhong L, Duan Z, Zhang Y, Zhu Y, Characterization of three H5N5 and one H5N8 highly pathogenic avian influenza viruses in China. Vet Microbiol. 2013;163:351–7. 10.1016/j.vetmic.2012.12.025 23375651

[4] Munch M, Nielsen LP, Handberg KJ, Jørgensen PH. Detection and subtyping (H5 and H7) of avian type A influenza virus by reverse transcription-PCR and PCR-ELISA. Arch Virol. 2001;146:87–97. 10.1007/s007050170193 11266220

[5] Hoffmann E, Stech J, Guan T, Webster RG, Perez DR. Universal primer set for the full-length amplification of all influenza A viruses. Arch Virol. 2001;146:2275–89. 10.1007/s007050170002 11811679

[6] World Organisation for Animal Health. Manual of diagnostic tests and vaccines for terrestrial animals, 2013. Avian influenza. Chapter 2.3.4 [cited 2014 Mar 10]. http:// www.oie.int/en/international-standard-setting/terrestrial-manual/ access-online/.

[7] World Organisation for Animal Health. OIE 14668, January 17, 2014, Country: Korea [cited 2014 Mar 10]. http://www.oie.int/wahis_2/temp/reports/en_imm_0000014668_20140120_160850.pdf

[8] Stevens J, Blixt O, Tumpey TM, Taubenberger JK, Paulson JC, Wilson IA. Structure and receptor specificity of the hemagglutinin from an H5N1 influenza virus. Science. 2006;312:404–10. 10.1126/science.1124513 16543414

[9] Orozovic G, Orozovic K, Lennerstrand J, Olsen B. Detection of resistance mutations to antivirals oseltamivir and zanamivir in avian influenza A viruses isolated from wild birds. PLoS ONE. 2011;6:e16028. 10.1371/journal.pone.0016028 21253602 PMC3017088

[10] Gu M, Zhao G, Zhao K, Zhong L, Huang J, Wan H, Novel variants of clade 2.3.4 highly pathogenic avian influenza A(H5N1) viruses, China. Emerg Infect Dis. 2013;19:2021–4. 10.3201/eid1912.130340 24274396 PMC3840869

